# Alarming rates of antimicrobial resistance and fungal sepsis in outborn neonates in North India

**DOI:** 10.1371/journal.pone.0180705

**Published:** 2018-06-28

**Authors:** Mamta Jajoo, Vikas Manchanda, Suman Chaurasia, M. Jeeva Sankar, Hitender Gautam, Ramesh Agarwal, Chander Prakash Yadav, Kailash C. Aggarwal, Harish Chellani, Siddharth Ramji, Monorama Deb, Rajni Gaind, Surinder Kumar, Sugandha Arya, Vishnubhatla Sreenivas, Arti Kapil, Purva Mathur, Reeta Rasaily, Ashok K. Deorari, Vinod K. Paul, Investigators of the Delhi Neonatal Infection Study (DeNIS) collaboration, New Delhi, India

**Affiliations:** 1 Department of Pediatrics, Chacha Nehru Bal Chikitsalaya, New Delhi, India; 2 Department of Microbiology, Chacha Nehru Bal Chikitsalaya, New Delhi, India; 3 Department of Pediatrics, All India Institute of Medical Sciences, New Delhi, India; 4 Department of Biostatistics, All India Institute of Medical Sciences, New Delhi, India; 5 National Institute of Malaria Research, New Delhi, India; 6 Department of Pediatrics, Vardhman Mahaveer Medical College and Safdarjung Hospital, New Delhi, India; 7 Department of Pediatrics, Maulana Azad Medical College and LNJP Hospital, New Delhi, India; 8 Department of Microbiology, Vardhman Mahaveer Medical College and Safdarjung Hospital, New Delhi, India; 9 Department of Microbiology, Maulana Azad Medical College and LNJP Hospital, New Delhi, India; 10 Department of Microbiology, All India Institute of Medical Sciences, New Delhi, India; 11 Department of Laboratory Medicine, JPNA Trauma Centre, AIIMS, New Delhi, India; 12 Division of Reproductive Health & Nutrition, Indian Council of Medical Research (ICMR), New Delhi, India; Yale School of Public Health, UNITED STATES

## Abstract

**Background:**

There is a paucity of data on the epidemiology of sepsis in outborn neonates being referred to level-3 units in low- and middle-income countries (LMIC). The objective of the present study was to evaluate the prevalence of sepsis and outcomes of outborn neonates with sepsis, and to characterize the pathogen profile and antimicrobial resistance (AMR) patterns of common isolates in them.

**Methods:**

In this prospective observational cohort study (2011–2015), a dedicated research team enrolled all neonates admitted to an outborn level-3 neonatal unit and followed them until discharge/death. Sepsis work-up including blood culture(s) was performed upon suspicion of sepsis. All the isolates were identified and tested for antimicrobial susceptibility. Gram-negative pathogens resistant to any three of the five antibiotic classes (extended-spectrum cephalosporins, carbapenems, aminoglycosides, fluoroquinolones, and piperacillin-tazobactam) were labeled multi-drug resistant.

**Results:**

Of the total of 2588 neonates enrolled, culture positive sepsis and total sepsis–i.e. culture positive and/or culture negative sepsis–was diagnosed in 13.1% (95% CI 11.8% to 14.5%) and 54.7% (95% CI 52.8% to 56.6%), respectively. The case fatality rates were 23.4% and 11.0% in culture-positive and total sepsis, respectively. Sepsis accounted for two-thirds of total neonatal deaths (153/235, 63.0%). Bacterial isolates caused about three-fourths (296/401; 73.8%) of the infections. The two common pathogens–*Klebsiella pneumoniae* (n = 50, 12.5%) and *Acinetobacter baumannii* (n = 46, 11.5%)–showed high degree of multi-drug resistance (78.0% and 91.3%, respectively) and carbapenem resistance (84.0% and 91.3%, respectively). About a quarter of infections were caused by Candida spp. (n = 91; 22.7%); almost three-fourths (73.7%) of these infections occurred in neonates born at or after 32 weeks’ gestation and about two-thirds (62.1%) in those weighing 1500 g or more at birth.

**Conclusions:**

In this large outborn cohort, we report high burden of sepsis, high prevalence of systemic fungal infections, and alarming rates of antimicrobial resistance among bacterial pathogens.

## Introduction

In most low- and middle- income countries (LMICs), a large number of sick neonates born elsewhere get admitted to neonatal intensive care units (NICU) for advanced care. Compared to ‘inborn’ neonates who are born *and* admitted in the same hospital, these ‘outborn’ or ‘extramural’ neonates often have worse outcomes. [1–5]

Sepsis is one of the commonest diagnoses at admission in outborn neonates. In contrast to inborn neonates, sepsis in outborn neonates has several distinct features [6, 7]: first, the source of infection can be either community- or hospital- acquired depending upon the place of birth and prior hospitalization. Second, for obvious reasons, the denominator for estimating the risk of sepsis cannot be live births. Third, one cannot accurately estimate the *incidence* of sepsis because many might already be harboring infection at the time of admission. Finally, outcomes of neonates with sepsis are heterogeneous because of differences in source of infection, AMR, timing of referral, and level of sickness at admission.

Despite the peculiar epidemiology, there is a paucity of high quality data on sepsis among outborn neonates. The National Neonatal-Perinatal Database (NNPD) network of India that provided data on 3831 and 11026 outborn neonates respectively in years 2000[8] and 2002–03[9] highlighted a different morbidity and mortality profile in these neonates. Subsequently, there have been a few published studies, largely based on routinely collected clinical and laboratory data, with the inherent limitations of quality. [10–15] The paucity of reliable data on sepsis in outborn neonates has compromised benchmarking of practices, stymied research investments, and undermined potential policies for change. In the present study, we report prospectively collected data over a three-year period from an exclusively outborn NICU.

## Methods

### Study setting and population

The Delhi Neonatal Infection Study (DeNIS) collaboration, established in 2010, is a network of investigators of four tertiary care academic units namely, Vardhaman Mahavir Medical College, Maulana Azad Medical College, All India Institute of Medical Sciences (AIIMS; the co-ordinating centre), and Chacha Nehru Bal Chikitasalaya (CNBC; 20 bedded outborn unit with annual turnover of 700 infants) in Delhi. In the current paper, we report the data of the outborn cohort from CNBC; data on the inborn cohort from the other three hospitals has been published elsewhere. [16]

All neonates requiring admission in NICU at CNBC from July 2011 to February 2015 were enrolled in the study, after obtaining informed consent from one of the parents. Neonates who required re-admission were excluded. A dedicated research team prospectively recorded the demographic details (obtained by eliciting history from the mother/father) and details of previous hospitalization (usually obtained from the referral note) in a pre-designed case record form (CRF).

### Sepsis work-up

All enrolled neonates were monitored for signs of sepsis at admission and later on a daily basis until discharge or death by the clinical team. If a neonate was suspected to have sepsis based on presence of perinatal risk factors and/or symptoms and signs delineated in Young Infant Study algorithm [17], the research team performed sepsis work up including blood/cerebrospinal fluid/other sterile fluid cultures and sepsis screen (S1 File: Table A). The cultures were performed as per the standard protocol (S1 File: Panel A; [16]), following which the clinical team initiated antimicrobials as per the unit policy (first line: ampicillin and gentamicin for those suspected and not exposed to antibiotics, cefotaxime and amikacin if exposed to antibiotics; second line: piperacillin-tazobactam and netilmicin). The research team recorded the relevant information of sepsis workup in CRFs.

### Microbiology processing

A dedicated microbiology research team processed the culture samples as per the standard protocol. For blood culture, a blood volume of 0.5–1 mL was drawn in Bactec Peds Plus (Becton Dickinson, USA) vial. The blood sample in Bactec vial was incubated in Bactec 9120/ Bactex FX 200 machines (Becton Dickinson, USA). The specimens indicating growth were sub-cultured on ready prepared 5% sheep blood agar and chocolate agar (bioMerieux, France), and in-house prepared MacConkey agar (Oxoid, UK). In cases with suspected fungemia, additionally, blood pecimens were collected in Bactec Myco F Lytic (Becton Dickinson, USA) bottle. On gram staining of the positive beeped bottle, additional inoculations were made on yeast chrome agar to rule out multiple yeast infections. The pathogens were identified and antimicrobial susceptibility testing (AST) was performed using Vitek-2 compact system (bioMérieux, France). AST results were interpreted as per Clinical and Laboratory Standards Institute (CLSI) guidelines as susceptible, intermediate (I), or resistant (R). [18–20]

The Gram-negative pathogens’ resistance profiles were also categorized based on resistance to various antimicrobial classes namely, (i) extended-spectrum cephalosporins (any two of ceftazidime, ceftriaxone, or cefotaxime), (ii) aminoglycosides (any one of gentamicin, amikacin, or netilmicin), (iii) carbapenem (imipenem or meropenem), (iv) fluoroquinolone (ciprofloxacin), and (v) piperacillin+tazobactam. If resistance to any three of the five specified classes were detected, the pathogen was labeled multidrug resistant (MDR).

### Outcomes and definitions

Two senior pediatricians (consultants) prospectively assigned the diagnosis of sepsis based on the clinical course, sepsis screen results, and culture reports using the definitions adapted from National Healthcare Safety Network (NHSN) (Table 1 and S1 File: Table A).[21,22] The causes of death were assigned prospectively by the clinical and research teams.

**Table 1:** Definitions used in the study*.

• Culture-positive sepsis
Isolation of a recognized pathogen from blood/CSF/other body fluids in neonates suspected to have sepsis based on clinical features or perinatal risk factors, and the neonate had received appropriate antibiotic therapy; In case of coagulase negative staphylococci (CoNS), culture-positive sepsis was labeled only if the clinical course was suggestive of sepsis and appropriate antibiotic therapy was given
• Culture-negative sepsis
Clinical course suggestive of sepsis OR positive sepsis screen but no pathogen isolated, with appropriate treatment for sepsis given
• Total sepsis
Neonates with culture-positive sepsis and/or culture-negative sepsis
• Meningitis
Should fulfill *ANY ONE* of the following criteria (i-ii) (i) CSF culture is positive *AND* Baby has at least 1 of the signs or symptoms (ii) If culture is negative, *all* of the following: Any one of the clinical sign/symptoms listed above *AND* [Any one of a–b: a) Positive CSF examination with neutrophilic leukocytosis, with or without low sugar (below 50% of plasma glucose level) and high-protein content, b) Positive gram stain for CSF] *AND*, physician institutes appropriate antimicrobial therapy (when diagnosis is made antemortem)
• Systemic fungal infections
Should fulfill *ANY ONE* of the following: (i) Blood culture positive for yeasts AND physician institutes appropriate therapy (ii) ≥2 of the risk factors^#^ AND presence of budding yeast/hyphae in either urine or cerebrospinal fluid AND physician institutes appropriate therapy
• Second (new) episode of sepsis
Second (new) episode of sepsis was considered when it was suspected after 48 hours of discontinuation of appropriate antibiotic therapy.

*Detailed definitions provided in S1 File: Table A

^#^ Risk factors include gestational age <32 weeks, previous fungal colonization (especially of the gastrointestinal tract), presence of central venous catheters, prior use of parenteral nutrition and lipid emulsions, duration of intubation >7 days, duration of hospitalization >7 days, shock or coagulopathy, exposure to >2 antibiotics or any of third-generation cephalosporins, systemic corticosteroids, H_2_ blockers or theophyllines

To examine the differences in pathogen profile and their AMR in community versus hospital acquired sepsis, we arbitrarily categorized neonates with culture-positive sepsis into four groups (S1 File: Table B): (i) ‘community acquired infection’ (CAI), (ii) ‘possibly community acquired infection’ (pCAI), (iii) ‘possibly healthcare-associated infection’ (pHAI) and (iv) ‘healthcare-associated infection’ (HAI). The CAI group included neonates who were born at home, had no prior admission in a health facility, and were diagnosed to have sepsis within 48 hours of admission to the study hospital. The pCAI group included neonates who were born at any health facility and discharged to home but were later admitted in the study hospital after 7 days of life and diagnosed to have sepsis within 48 hours of admission. The pHAI group included a) neonates similar to pCAI but were diagnosed to have sepsis *within* 7 days of postnatal age and b) neonates with prior history of hospitalization in other health facilities and were diagnosed to have sepsis within 48 hours’ admission to the study hospital. The fourth group, HAI, included neonates diagnosed to have sepsis after 48 hours of admission in the study hospital, irrespective of the place of birth or prior admission in any health facility.

### Data management and quality assurance

The research physician checked the CRFs daily for accuracy while the senior investigators at CNBC and at the coordinating center cross-checked them on a weekly basis. Data was entered in duplicate into an online database developed in Visual Basics as front-end and MS SQL server as back-end with inbuilt range and logical checks. Detailed quality assurance system for clinical and laboratory procedures as well as data management system were put in place (S1 File: Table C); a dry run was carried out for four weeks before rolling-out the study. The microbiology laboratory at CNBC has robust internal quality assurance program and had participated in National level external quality assurance scheme (EQAS). Additionally, we instituted study-specific EQAS (S1 File: Panel B), wherein identification and AST results for 10% of isolates were cross-checked at the coordinating center.

### Statistical analysis

Statistical analysis was performed using Stata 11·2 (StataCorp, College Station, TX, USA). Prevalence of sepsis was calculated by dividing the number of neonates with sepsis by total number of NICU admissions.

### Consent and ethics clearance

The study was approved by the Institutional Ethics Committee of CNBC and AIIMS. Written informed consent was taken from the parents of enrolled neonates.

## Results

**Fig 1:**
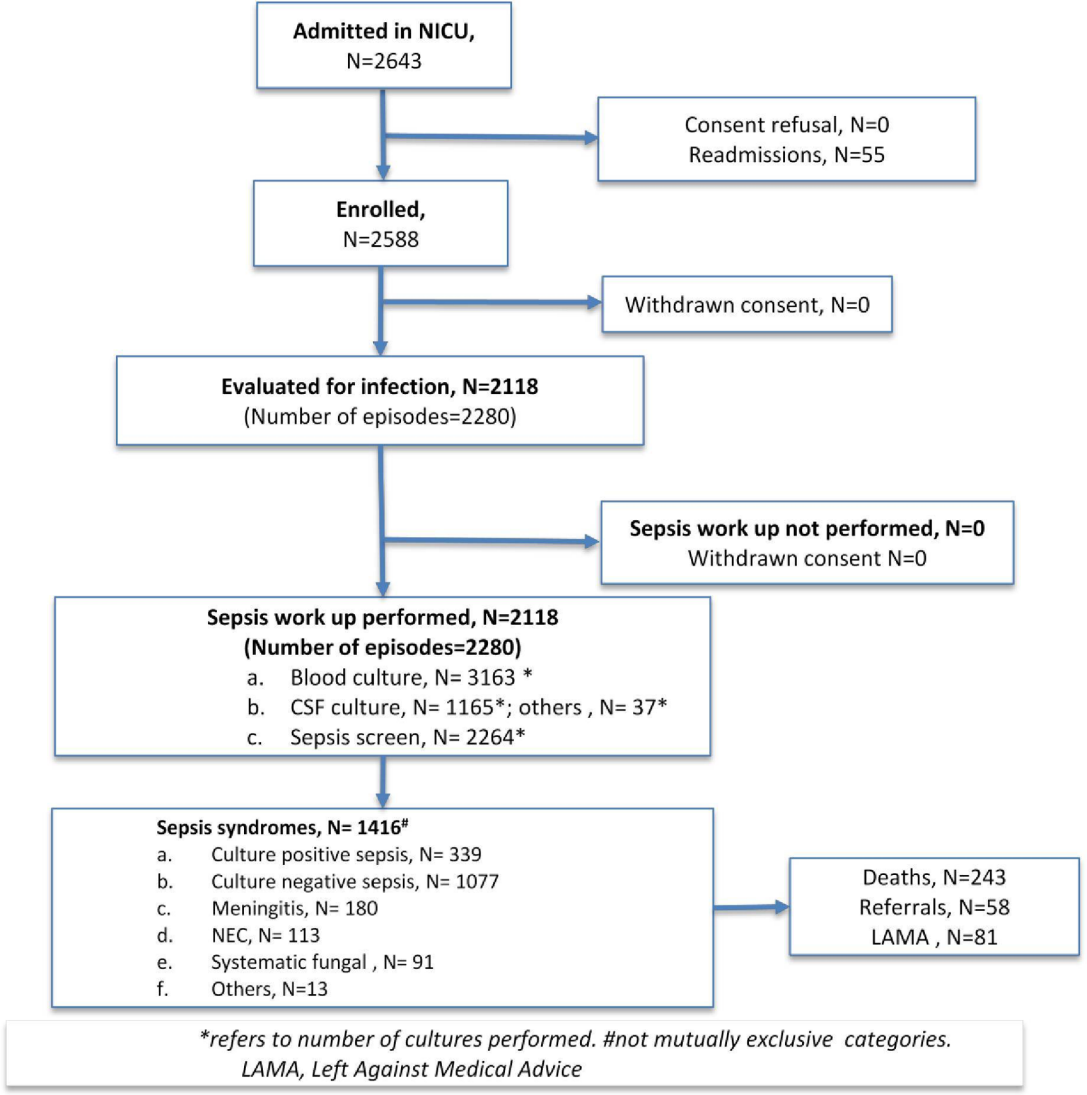
Study flow.

A total of 2588 of 2643 neonates admitted from July 2011 to February 2015 were enrolled, after excluding the re-admissions (n = 55) (Fig 1).

The mean birth weight and gestation were 2204 g and 35.4 weeks, respectively (Table 2). Over four-fifths of neonates were born at or after 32 weeks (2263/2583) and had a birth weight of 1500 g or more (1674/2058) (S1 File: Table D). About two-thirds of enrolled neonates were males (65%). Two-thirds were admitted within the first week of life–nearly a quarter within 24 h. One-fifth of neonates was born at home–most were delivered by traditional birth attendants (Table 2). Around one-third received unhygienic cord practices and prelacteal feeds. Over a third (38.0%) were referred after being admitted at another health facility–mostly private hospitals–after a median (IQR) duration of stay of 2 (1 to 7) days. Most of these neonates (83.8%) had received antibiotics for a median duration of 3 (1 to 6) days during prior hospitalization, the common antibiotics being amikacin, cefotaxime, meropenem, and piperacillin-tazobactam (Table 2).

**Table 2:** Demographic characteristics of enrolled infants.

Items	Values (n = 2588)
Birth weight, *g* (n = 2058)	2204±731
Gestational age, week (n = 2583)	35.4±2.8
Male gender	1680 (64.9%)
Age at admission, days	5 (2–11)
**Maternal details**	
• Urinary tract infection in last trimester	208/2287 (9.09%)
• Fever within 7 days prior to delivery	211/2303 (9.2%)
• Antibiotics within 7 days prior to delivery	142 (5.5%)
• Antenatal steroids (in <35 wk gestation; n/N)	102/701 (14.5%)
• Vaginal examinations (≥3)	702/2281 (30.8%)
• Rupture of membranes (>18h)	397/2577 (15.4%)
• Meconium stained liquor	364/2571 (14.2%)
• Foul smelling liquor	53/2550 (2.1%)
**Birth details**	
• Caesarean delivery	575/2580 (22.3%)
• Home delivery	550/2588 (21.2%)
• Delivery by traditional birth attendant	456/2306 (19.8%)
• Did not cry at birth	643/2577 (24.9%)
• Resuscitation at birth	182/2273 (8.01%)
• Unhygienic cord practices (application of oil, cow dung, etc.)	879/2544 (34.5%)
• Pre-lacteal feeds	682/2307 (29.6%)
**Previous hospitalization of the neonate (n = 984; 38.0%)**	
Healthcare facility	
• Primary/secondary level government hospital	56 (5.7%)
• Tertiary level government hospital	198 (20.1%)
• Private hospital	730 (74.2%)
Duration of stay, days	2 (1 to 7)
Mechanical ventilation	271/984 (27.5%)
Antibiotic therapy	825/984 (83.84%)
Duration of antibiotic therapy, days	3 (1 to 6)
Type of antibiotics	
• Amikacin	598 (72.5%)
• Cefotaxime	446 (54.1%)
• Meropenem	139 (16.8%)
• Piperacillin-tazobactam	116 (14.1%)
• Colistin	11 (1.3%)
**Therapeutic modalities at study hospital (n = 2306)**	
• Intravenous fluids	1950 (84.6%)
• Parenteral nutrition	1415 (61.4%)
• Central catheters*	820 (35.6%)
• Intermittent mandatory ventilation	653 (28.3%)
• Blood/plasma transfusion	461 (20.0%)
• Continuous positive airway pressure (CPAP)	319 (13.8%)
• Corticosteroids	310 (13.4%)
• Antibiotic therapy	2243/2588 (86.7%)
• Duration of antibiotic therapy	6 (4 to 12)
• Duration of NICU stay, days	6 (3 to 12)

Data expressed as no (%), median (IQR) or mean±SD

*include umbilical venous/arterial catheter or peripherally inserted central catheter

### Prevalence of sepsis

Overall, we suspected 2280 episodes of sepsis in 2118 (81.8%) neonates and processed 4365 cultures (Table 3; S1 File: Tables E-G). Sepsis was diagnosed in 1416 neonates (54.7%; 95% CI 52.8 to 56.6) and in 1458 episodes. A total of 339 neonates (13.1%; 95% CI 11.8 to 14.5) had culture-positive sepsis. Only few neonates– 28 and 7 –had two and three episodes of total sepsis, respectively. Meningitis, necrotizing enterocolitis, and systemic fungal infections were diagnosed in 180 (7.0%), 113 (4.4%), and 91 (3.5%) neonates, respectively (Table 3).

**Table 3:** Outcomes of enrolled neonates (n = 2588).

Items	Values, n (%)
**Total sepsis**	1416 (54.7%)
**Culture positive sepsis**	339 (13.1%)
*Community acquired infection (CAI)* ^#^	*48 (14*.*2%)*
*Possibly community acquired infection (pCAI)* ^#^	*41 (12*.*1%)*
*Possibly healthcare-associated infection (pHAI)* ^#^	*233 (68*.*7%)*
*Healthcare-associated infection (HAI)* ^#^	*17 (5*.*0%)*
**Culture negative sepsis**	1077 (41.6%)
**Meningitis**	180 (7.0%)
**Necrotizing enterocolitis**	113 (4.4%)
**Systemic fungal infection**	91 (3.5%)
Others*	13 (0.5%)
**Diagnosis of sepsis** (since time of admission)	
• Within 24 hours	1320 (93.6%)
• 25 to 48 hours	22 (1.6%)
• 49 to 72 hours	9 (0.6%)
• 73 hours to 7 days	11 (0.7%)
• 8 days or more	47 (3.3%)
**All-cause mortality**	243 (9.4%)
**Primary cause of death**	
• Infections	153 (62.9%)
• Prematurity	37 (15.2%)
• Perinatal asphyxia	9 (3.7%)
• Malformations	18 (7.4%)
• Others	26 (10.7%)
**Case fatality rates**, n (%)	
• Total sepsis	153/1416 (10.8%)
• Culture positive sepsis	78/339 (23.0%)
○ CAI	*12/48 (25*.*0%)*
○ pCAI	*8/41 (19*.*5%)*
○ pHAI	*53/233 (22*.*7%)*
○ HAI	*5/17 (29*.*4%)*
• Culture negative	73/1077 (6.8%)
• Meningitis	22/180 (12.2%)
• Necrotizing enterocolitis	20/113 (17.7%)
• Systemic fungal infection	20/90 (22.2%)

Data expressed as no (%)

^#^Refer to text for definitions (S1 File: Table B); mutually exclusive groups since only one baby had 2 episodes of culture positive sepsis.

See S1 File: Tables E-G for additional details.

*Bone/joint infection (n = 9), urinary tract infection (n = 4)

### Profile of pathogens

A total of 401 pathogens were isolated; bacterial pathogens accounted for about three-fourths. There was a predominance of Gram-negative isolates (52.9%; Table 4); the common isolates were *Klebsiella pneumoniae* (12.5%), *Acinetobacter baumannii* (11.5%), *Escherichia coli* (8.0%), and *Enterobacter cloacae* (5.7%). The common Gram-positive pathogens were *Staphylococcus aureus* (4.7%), *Staphylococcus epidermidis* (4.2%), *Staphylococcus hemolyticus* (3.2%), and *Enterococcus faecium* (3.0%). A quarter of isolates were fungi, predominantly comprising of *Candida tropicalis* (5.0%), *Candida albicans* (5.0%), and *Candida parapsilosis* (4.5%). Group B streptococci were isolated in only two neonates (Table 4).

**Table 4:** Profile of clinical isolates*.

	CAI (n = 58)	pCAI (n = 49)	pHAI (n = 275)	HAI (n = 19)	Overall (n = 401)
**Gram-negative**					
*Acinetobacter baumannii*	8 (13.8%)	2 (4.1%)	36 (13.1%)	0	46 (11.5%)
*Klebsiella pneumoniae*	6 (10.3%)	4 (8.2%)	39 (14.2%)	1 (5.3%)	50 (12.5%)
*Escherichia coli*	9 (15.5%)	4 (8.2%)	17 (6.2%)	2 (10.5%)	32 (8.0%)
*Enterobacter cloacae*	3 (5.2%)	4 (8.2%)	16 (5.8%)	0	23 (5.7%)
*Pseudomonas aeruginosa*	0	0	1 (0.4%)	0	1 (0.2%)
*Burkholderia cepacia*	1 (1.7%)	1 (2.0%)	10 (3.6%)	1 (5.3%)	13 (3.2%)
*Klebsiella oxytoca*	0	0	5 (1.8%)	0	5 (1.2%)
**Gram-positive**					
*Staphylococcus aureus*	5 (8.6%)	6 (12.2%)	6 (2.2%)	2 (10.5%)	19 (4.7%)
*Staphylococcus epidermidis*	4 (6.9%)	2 (4.1%)	9 (3.3%)	2 (10.5%)	17 (4.2%)
*Staphylococcus hemolyticus*	1 (1.7%)	3 (6.1%)	9 (3.3%)	0	13 (3.2%)
*Staphylococcus hominis*	2 (3.4%)	2 (4.1%)	3 (1.1%)	0	7 (1.7%)
*Streptococcus agalactiae*	1 (1.7%)	0	1 (0.4%)	0	2 (0.5%)
*Enterococcus faecium*	1 (1.7%)	5 (10.2%)	6 (2.2%)	0	12 (3.0%)
**Fungi**					
*Candida albicans*	0	0	18 (6.5%)	2 (10.5%)	20 (5.0%)
*Candida tropicalis*	1 (1.7%)	1 (2.0%)	15 (5.5%)	3 (15.8%)	20 (5.0%)
*Candida krusei*	1 (1.7%)	2 (4.1%)	13 (4.7%)	0	16 (4.0%)
*Candida parapsilosis*	1 (1.7%)	3 (6.1%)	13 (4.7%)	1 (5.3%)	18 (4.5%)
*Candida pelliculosa*	0	1 (2.0%)	8 (2.9%)	0	9 (2.2%)
*Candida glabrata*	0	1 (2.0%)	7 (2.5%)	0	8 (2.0%)
**Others^#^**	14 (24.1%)	8 (16.3%)	43 (15.6%)	5 (26.3%)	70 (17.5%)

CAI, Community acquired Infection; pCAI, possibly community acquired Infection; pHA, possibly healthcare-associated infection; HAI, healthcare-associated infection

*only 26 isolates were obtained from sterile fluids other than blood (like CSF, urine)

^#^Include *Achromobacter xylosoxidans* (n = 2); *Acinetobacter lwoffii (n = 3); Aeromonas hydrophilia* (n = 1)*; Aerococcus viridans* (n = 2); *Brevundimonas diminuta* (n = 1); *Brevundimonas* spp (n = 1)*; Citrobacter freundii* (n = 3)*; Cryptococcus laurentii* (n = 3)*; Enterobacter sakazaki* (n = 2)*; Enterococcus fecalis* (n = 2)*; Hemophilus influenzae* (n = 1)*; Kocuria kristinae* (n = 1)*; Kodamaea ohmeri* (n = 2)*; Leuconostoc pseudomesenteroides* (n = 1)*; Morganella morganii* (n = 1); Non fermenting gram negative organism (n = 1); Other *Burkholderia* spp. *(n = 1);* Other *Candida* spp. (n = 10); Other CoNS (n = 6); Other enterococci (n = 5); Other *Pseudomonas* spp. (n = 3); Other *Streptococci* (n = 1); *Pantoea* spp (n = 4)*; Proteus mirabilis* (n = 2)*; Proteus vulgaris* (n = 1)*; Salmonella typhi* (n = 1)*; Serratia marcescens* (n = 1)*; Sphingomonas paucimobilis* (n = 1); *Stenotrophomonas maltohilia* (n = 1); *Streptococcus pneumoniae* (n = 4); *Streptococcus pyogenes* (n = 2).

Of the sub-groups of neonates with culture positive sepsis, there was a predominance of Gram-negative organisms—*E coli* (n = 9); *A baumannii* (n = 8); and *K pneumoniae* (n = 6) in neonates with CAI (Table 4). Among those with pCAI, Gram-positive organisms (*S aureus*, n = 6 and *E faecium*, n = 5) followed by enterobacteriacae (*K pneumoniae*, n = 4; *E coli*, n = 4; and *E cloacae*, n = 4) were the common isolates. In neonates with pHAI, there was a predominance of Gram-negative organisms (*K pneumoniae*, n = 39, 14.2% and *A baumannii*, n = 36, 13.1%) and Candida (*C albicans*, n = 18, 16.5% and *C tropicalis*, n = 15, 5.5%) while in those with HAI, Candida spp. were predominant organisms (*C tropicalis*, n = 3 and *C albicans*, n = 2).

### Antimicrobial resistance

Most bacterial isolates revealed high degree of AMR (S1 File:Tables H-I), even to “rescue” antibiotics like carbapenems, vancomycin, linezolid, teicoplanin and colistin. Carbapenem resistance in common Gram-negative pathogens–*Klebsiella pneumoniae*, *Acinetobacter baumannii*, *Escherichia coli*, and *Enterobacter cloacae–*was 72.1%, 90.7%, 30.0% and 58.3%, respectively (Table 5). MDR in these isolates was 79.5%, 90.7%, 56.7% and 66.7%, respectively. High rates of methicillin resistance were noted in *Staphylococcus aureus* (31.6%), *Staphylococcus epidermidis* (88.2%), and *Staphylococcus hemolyticus* (100%). Vancomycin resistance was found in 41.7% of enterococci (Table 5).

**Table 5:** Antimicrobial resistance (AMR) and case fatality rates among common pathogens by their AMR pattern.

Pathogens	Antimicrobial class	Resistance	CFR in culture positive sepsis due to	
			Resistant pathogens	Sensitive pathogens
**Gram negative**				
*Klebsiella pneumonia* (n = 50)	ES cephalosporins	42/50 (84.0%)	9/42 (21.4%)	1/8 (12.5%)
	Carbapenems	35/50 (70.0%)	8/35 (22.9%)	2/15 (13.3%)
	Aminoglycosides	42/50 (84.0%)	10/42 (23.8%)	0/8
	MDR	39/50 (78.0%)	10/39 (25.6%)	0/11
*Acinetobacter baumannii* (n = 46)	ES cephalosporins	42/46 (91.3%)	16/42 (38.1%)	1/4 (25.0%)
	Carbapenems	42/46 (91.3%)	17/42 (40.5%)	0/4
	Aminoglycosides	42/46 (91.3%)	17/42 (40.5%)	0/4
	MDR	42/46 (91.3%)	17/42 (40.5%)	0/4
*Escherichia coli* (n = 32)	ES cephalosporins	24/32 (75.0%)	9/24 (37.5%)	4/8 (50.0%)
	Carbapenems	11/32 (34.4%)	5/11 (45.4%)	8/21 (61.9%)
	Aminoglycosides	17/32 (53.1%)	9/17 (52.9%)	4/15 (26.7%)
	MDR	18/32 (56.2%)	9/18 (50.0%)	4/14 (28.6%)
*Enterobacter cloacae* (n = 23)	ES cephalosporins	16/23 (69.6%)	2/16 (12.5%)	1/7 (14.3%)
	Carbapenems	13/23 (56.5%)	2/13 (15.4%)	1/10 (10.0%)
	Aminoglycosides	16/23 (69.6%)	2/16 (12.5%)	1/7 (14.3%)
	MDR	15/23 (65.2%)	2/15 (13.3%)	1/8 (12.5%)
**Gram positive**				
*Staphylococcus aureus* (n = 19)	Meticillin	6/19 (31.6%)	2/6 (33.3%)	1/13 (7.7%)
	Vancomycin	1/19 (5.3%)	0/1	3/18 (16.7%)
*Staphylococcus epidermidis* (n = 17)	Meticillin	15/17 (88.2%)	2/15 (13.3)	0/2
	Vancomycin	0/17	-	2/17 (11.8%)
*Staphylococcus hemolyticus* (n = 13)	Meticillin	12/12 (100%)	3/12 (25.0%)	-
	Vancomycin	0/12	-	3/12 (25.0%)
*Staphylococcus hominis* (n = 7)	Meticillin	7/7 (100%)	1/7 (14.3%)	-
	Vancomycin	0/7	-	1/7 (28.6%)
*Enterococcus faecium* (n = 12)	Meticillin	2/3 (66.7%)	0/2	1/1
	Vancomycin	5/12 (41.7%)	1/5 (20.0%)	2/7 (28.6%)

Data expressed in n/N (%);Carbapenems: Meropenem or imipenem; fluoroquinolones: ciprofloxacin; aminoglycosides: amikacin or netilmicin or gentamicin; ES cephalosporins (ESC); for details, refer to text; there are variations in denominators in each cell as antibiotics sensitivity testing for all drugs was not done

AMR pattern of most pathogens in the subgroups of culture positive sepsis were similar (Table 6). Among the four common Gram-negative bacilli, cumulative rates of multi-drug resistance were 65.4%, 71.4%, 78.7% and 66.0% while that of carbapenem resistance were 50.0%, 64.3%, 67.6% and 66.0% in CAI, pCAI, pHAI and HAI groups respectively (Table 6).

**Table 6:** Antimicrobial resistance of common Gram-negative isolates.

	*K pneumoniae* (n = 50)				*A baumannii* (n = 46)				*E coli* (n = 32)				*E cloacae* (n = 23)			
Antimicrobial classes	CAI (n = 6)	pCAI (n = 4)	pHAI (n = 39)	HAI (n = 1)	CAI (n = 8)	pCAI (n = 2)	pHAI (n = 36)	HAI (n = 0)	CAI (n = 9)	pCAI (n = 4)	pHAI (n = 17)	HAI (n = 2)	CAI (n = 3)	pCAI (n = 4)	pHAI (n = 16)	HAI (n = 0)
**ES cephalosporins**	4/ 6 (66.7)	4 /4 (100)	33/39 (84.6)	1 /1 (100)	7/8 (87.5)	2 /2 (100)	33 /36 (91.7)	**-**	6 /9 (66.7)	3/ 4 (75)	13 /17 (76.5)	2/2 (100)	2/3 (66.7)	2/4 (50)	12/ 16 (75)	**-**
**Carbapenems**	4/ 6 (66.7)	4/ 4 (100)	22 /39 (66.7)	1 /1 (100)	7/8 (87.5)	2/ 2 (100)	33 /36 (91.7)	**-**	0 /9 (0)	1 /4 (25)	9 /17 (52.9)	1/2 (0)	2/3 (66.7)	2 /4 (50)	9 /16 (56.2)	**-**
**Aminoglycosides**	4/ 6 (66.7)	4/ 4 (100)	33/39 (84.6)	1/ 1 (100)	7/8 (87.5)	2 /2 (100)	33 /36 (91.7)	**-**	4 /9 (44.4)	2 /4 (50)	10 /17 (58.8)	1/2 (0)	2/3 (66.7)	2/ 4 (50)	12 /16 (75)	**-**
**MDR**	4/ 6 (66.7)	4 /4 (100)	30/ 39 (76.9)	1 1 (100)	7/8 (87.5)	2 2 (100)	33 /36 (91.7)	**-**	4/ 9 (44.4)	2 /4 (50)	11 /17 (64.7)	1/2 (0)	2/3 (66.7)	2 4 (50)	11/ 16 (68.8)	**-**
**Colistin**	0 /6	0 /4	0 /39	0 /1	0 /8	0/ 2	1 /35 (2.8)	**-**	0 /9	0/ 4	0/16	0/ 2	0 /3	0 /4	0 /16	**-**

Data expressed as n (%)

Carbapenems: Meropenem or imipenem; fluoroquinolones: ciprofloxacin; aminoglycosides: amikacin or netilmicin or gentamicin; ES cephalosporins (ESC): for details, refer to text; CAI, Community acquired Infection; pCAI, possibly community acquired Infection; pHAI, possibly healthcare associated infection; HAI, healthcare associated infection

### Systemic fungal infections

Among the 90 neonates diagnosed with fungal infections, the mean (SD) birth weight and gestation were 1751 (698) g and 33.8 (4) weeks, respectively. Around two-thirds of these neonates were born at or after 32 weeks of gestation (n = 67, 73.3%) and had a birth weight of 1500 g or more (n = 49, 61.5%). The median (IQR) age at admission was 7.2 (3.7–15.6) days. Most (90.2%) were diagnosed within 12 hours of admission to index hospital (median [IQR] of admission-to-detection interval 0 [0–1] h). Three-fourths of neonates (n = 66, 73.3%) were hospitalized previously, the median duration of stay being 6.5 (4–14) days. Almost all of them (n = 62) had received broad-spectrum antibiotics including cephalosporins (50.0%), meropenem (32.3%), piperacillin-tazobactam (16.1%), and colistin (9.7%). Over 40% (27/62) had received mechanical ventilation (median [IQR]: 5 [2–8] days) during their prior hospital stay.

Only one of the tested isolates (*Candida sphaerica*) was resistant to fluconazole and voriconazole. Four isolates (*Candida krusei* (n = 2) and *Candida guilliermondii* (n = 2)) were resistant to Amphotericin B (S1 File: Table J).

### Outcomes

Sepsis was the most common cause of death, accounting for two thirds of total deaths (153/243; 63.0%; 95% CI 56.6 to 69.0; Table 3). The case fatality rates (CFR) of culture-positive and culture negative sepsis were 23.0% and 6.8%.; CFRs in the sub-groups of CAI, pCAI, pHAI, and HAI were 25.0%, 19.5%, 22.7%, and 29.4%, respectively. Systemic fungal infections also showed comparable CFR (22.0%). Amongst the common isolates, CFR was the highest for *Escherichia coli* (40.6%) followed by *Acinetobacter baumannii* (37.0%) and *Candida parapsilosis* (33.3%; S1 File: Table K). The CFR among multi-drug resistant pathogens were in general higher than ‘sensitive’ isolates (Table 5). Among the neonates with MDR sepsis, only half survived (74/131, 56.5%).

## Discussion

With over 2500 enrolled neonates, the present study is one of the largest studies on sepsis in outborn neonates from LMICs. It had a heterogeneous mix of neonates–one-fifth was born at home while two-fifths were referred from another health facility at different postnatal ages. The four major findings include (i) a very high prevalence of sepsis, which was also the foremost cause of in-hospital mortality; (ii) unusual rates of invasive fungal infections, with Candida spp. being the most commonly isolated pathogen; (iii) an alarmingly high degree of AMR among the bacterial isolates; and (iv) an unexpectedly high level of AMR even in community acquired infections.

More than half of the enrolled neonates (55%) had final diagnosis of sepsis, of whom a quarter had culture positive sepsis. Though the CFR was only 11% and 23% in total and culture-positive sepsis, respectively, sepsis accounted for two-thirds of deaths. The very high prevalence of sepsis, hitherto unreported, is indeed a cause of concern. The NNPD network–involving 10 leading outborn units in India–reported a prevalence of about 40%, a decade ago. [9] Rates of 53% in an older study from Malaysia and 27% from a recent 17-centers Canadian study have been reported among outborn *very low birth weight* (VLBW) neonates. [5, 7] The mean birth weight in our cohort was about 2200 g but still the prevalence of sepsis was much higher. In contrast, sepsis rates (of 11.5% to 16.5%) even in the highest-risk category of *preterm/VLBW* inborn neonates are much lower in the high-income countries. [23–27]

What are the possible reasons for such a high prevalence of sepsis in the present study? A selective referral bias of preterm neonates is unlikely to be an explanation because more than half of the neonates with sepsis were born at term gestation. Over-diagnosis of sepsis–a potential risk with a high prevalence of culture-negative sepsis–is also unlikely because the diagnosis was assigned prospectively based on the clinical course and investigations (sepsis screen was positive in 61% of neonates with culture-negative sepsis). The fact that most (94%) infections were diagnosed within 24 h of admission suggests that the neonates were already harboring infection at the time of admission in the study hospital (S1 File: Table F). This signifies that prior hospitalization is one of the strong risk factors of sepsis in the referred neonates, most (about 80%) of who came from private hospitals/nursing homes. It is possible that many, if not all, of these units have sub-optimal infection control practices [28] and irrational antibiotic policies. There is a definite need to optimize infection control practices and facilitate implementation of quality control measures in these units. [28–31] Among home-delivered neonates, the high rates of unhygienic cord practices and lack of breastfeeding might explain the high prevalence of sepsis.

More than a quarter of neonates with culture positive sepsis in the present study had systemic fungal infections. Studies from LMICs have seldom reported such predominance of fungal sepsis in outborn neonates. More striking is the case-mix of fungal sepsis–nearly two-thirds weighed 1500 g or more at birth while 70% were born at or after 32 weeks’ gestation. In contrast, systemic fungal infections in HICs are usually reported among preterm (born at or before 32 weeks’ gestation) VLBW neonates who are at risk following prolonged ventilation and parenteral nutrition, and antibiotic therapy with broad spectrum antibiotics.[22] Such risk factors are unlikely in more mature neonates. Still, a sizeable proportion (2.9%, 67/2263) of mature neonates developed fungal sepsis in the present study. As observed with infections in general, most (93%) cases of systemic fungal sepsis were diagnosed within 24 h of admission in study hospital. Interestingly, most of them were a week old and nearly three-fourths had history of prior hospitalization with uniform previous exposure to broad-spectrum antibiotics like third-generation cephalosporins (50%) or meropenem (32%). This again highlights the need to review antibiotic policies and establish antibiotic stewardship programs in referring hospitals.

We found an alarming prevalence of AMR including MDR (56% to 91%) amongst sepsis causing organisms. We used classes of higher-spectrum antibiotics (carbapenems, piperacillin–tazobactam), rather than the WHO-recommended first-line options [32] such as ampicillin, gentamicin, and cefotaxime [33,34] to define MDR. Still, we observed worse AMR profile. More importantly, the AMR rates in the outborn cohort were higher than that in inborn cohort of DeNIS collaboration. [16] For example, the prevalence of MDR in the isolates of *Acinetobacter*, *Klebsiella* spp., and *E coli* was 91%, 78%, and 56%, respectively in the outborn cohort whereas the corresponding figures were 82% (181/222), 54% (91/169), and 38% (52/139) in the inborn cohort. Among Gram-positive pathogens also, meticillin resistance was detected in 88% (15/17 of *S*. *epidermidis*) and 61% (85/140 of coagulase negative staphylococci) isolates in the outborn and inborn cohorts, respectively. In general, such high rates of AMR are also in line with recent reports from India and other LMICs. [35–39]

Interestingly, the profile of pathogens differed among the sub-groups–Enterobacteriacae members were the leading Gram-negative organisms in the CAI group, the Gram-positive pathogens dominated the pCAI group, both Gram-negative and fungi were prevalent in the pHAI group, while the Candida spp. emerged as the commonest isolate in HAI category. But the degree of AMR was almost comparable among all the categories (with the possible exception of *E coli*; Table 6). While it is possible that neonates in the pCAI group may have been colonized with resistant pathogens in the birthing hospitals due to unhygienic practices during childbirth [28], the more disturbing fact is the high level of AMR observed even among neonates with CAI group who were born at home and had no prior exposure to health facilities. This possibly indicates population level penetration of AMR-escalating factors like rampant use of antibiotics in health/other sectors. [39,40] Assuming that the AMR pattern of isolates from community-acquired infections (CAI and pCAI) are really representative of the true scenario in LMIC settings, the threat of ‘post-antibiotics era’ [39, 41, 42] does not appear to be imaginary any longer.

The major strength of our study includes high methodological rigor and prospective classification of sepsis. However, our study has a few limitations. First, the data is representative of a tertiary level outborn NICU with a high likelihood of referral bias—sicker and infected babies admitted more often. Second, the classification of pCAI and pHAI sepsis was, at best, arbitrary. There may be some degree of overlap between the two–for example, the former group included neonates who were discharged following safe hospital delivery but became symptomatic after seven days of life. It is possible that a few neonates stayed in their birth hospital for maternal indications for 5–6 days before getting discharged–they were more likely to have healthcare associated infections.

## Conclusions

In this study involving one of the largest prospective cohort of outborn neonates from India, we report high burden of sepsis related deaths, high rates of fungal infection in referred neonates and alarming rates of antimicrobial resistance among the bacterial isolates causing increased mortality, even among those of possible community origin. There is an urgent need to undertake measures aimed at prevention, and timely detection and referral of neonates with sepsis from community as well as hospitals. National programs need to implement antibiotic stewardship policies at utmost priority at various levels of health system in order to achieve reduction in IMR in line with the sustainable development goals (SDGs).

## Supporting information

S1 File Table A: Definitions used in the study.
Table B: Definition of groups.
Table C: Quality assurance measures.
Table D: Additional demographic details.
Table E: Birth weight and gestational age specific prevalence rates of infections.
Table F: Admission to diagnosis interval.
Table G: Outcomes compared by place of delivery.
Table H: AMR in gram positive pathogens.
Table I: AMR in gram negative pathogens.
Table J: Antimicrobial resistance pattern of fungal organisms.
Table K: Pathogen specific case fatality rates.
Panel A: Clinical SOP algorithm.
Panel B: External Quality Assurance Scheme (EQAS).
(DOCX)

S2 File Table A: STROBE checklist.
(DOCX)

S3 File List of authors and their contributions. (DOCX)
