# Supplementary material for: Alarming rates of antimicrobial resistance and fungal sepsis in outborn neonates in North India
Source: PLoS One. 2018 Jun 28;13(6):e0180705. doi: 10.1371/journal.pone.0180705 (PMC6023165; doi:10.1371/journal.pone.0180705)
Supplement: S1 File — Table A: Definitions used in the study. Table B: Definition of groups. Table C: Quality assurance measures. Table D: Additional demographic details. Table E: Birth weight and gestational age specific prevalence rates of infections. Table F: Admission to diagnosis interval. Table G: Outcomes compared by place of delivery. Table H: AMR in gram positive pathogens. Table I: AMR in gram negative pathogens. Table J: Antimicrobial resistance pattern of fungal organisms. Table K: Pathogen specific case fatality rates. Panel A: Clinical SOP algorithm. Panel B: External Quality Assurance Scheme (EQAS). (DOCX) [file pone.0180705.s001.docx]

### S1 File PLOSone “Outborn” manuscript

# Contents

| SN | Item | Page # |
| --- | --- | --- |
|  | Table A*:* Definitions used in the study | 3-4 |
|  | Table B Definition of groups | 5 |
|  | Table C: Quality assurance measures | 6 |
|  | Table D: Additional demographic details | 7 |
|  | Table E: Birth weight and gestational age specific prevalence rates of infections | 8 |
|  | Table F: Admission to diagnosis interval | 9 |
|  | Table G: Outcomes compared by place of delivery | 10 |
|  | Table H: AMR in Gram positive pathogens | 11 |
|  | Table I: AMR in Gram negative pathogens | 12 |
|  | Table J: Antimicrobial resistance pattern of fungal organisms | 13 |
|  | Table K: Pathogen specific case fatality rates | 14 |
|  | S1 Panel A: Clinical SOP algorithm | 15-16 |
|  | S1 Panel B: External Quality Assurance Scheme (EQAS) | 18 |
|  | References to S1 | 19 |

**Table A*:* Definitions used in the study (adapted from CDC-NHSN^1^)**

| Diagnosis | Working definitions | Notes/remarks |
| --- | --- | --- |
| Culture-positive sepsis (Laboratory-confirmed bloodstream infection) | Blood culture positive AND must meet ANY ONE of the following criteria:   1. True pathogen detected during sepsis work up*: Baby has a recognized pathogen cultured from 1 or more blood cultures and organism cultured is not related to an infection at another site   AND  Physician institutes appropriate treatment for septicemia   1. CoNS*: CoNS is cultured from 1 or more blood samples drawn on separate occasions (see “Notes”) and organism cultured is not related to an infection at another site   AND  Baby has at least 1 of the signs or symptoms enlisted (see “Notes”)  AND  Physician institutes appropriate treatment  (anti-staphylococcal penicillin or vancomycin)   1. Pathological evidence of sepsis on autopsy   *Other skin contaminants such as diphtheroids (*Corynebacterium* spp.), *Bacillus* (not *B anthracis*) spp., *Propionibacterium* spp., *Aerococcus* spp., *Micrococcus* spp.·were considered as contaminants  In addition, the following were considered as contaminants:   1. Organisms not fulfilling the definition of culture positive sepsis 2. Three or more organisms in a single culture | Any one of the following symptoms/signs:  Difficulty feeding  Convulsions  Movement only when stimulated  Diarrhea (watery stools)  Pus from umbilical stump  Discharge (purulent) from ear  Temperature (>37·5 °C or <36·5 °C)  Heart rate (>180 min or <100 min)  Respiratory rate (>60 min)  Severe chest in-drawing  Grunting  Apnea  CRT > 3 sec  Cyanosis  Lethargy/drowsiness  Bulging fontanel  Abdominal distension  Multiple (>10) skin pustules  Clinicians’ discretion- (such as neonate not looking well, need for augmentation of respiratory support, etc.)  Neonates were also subjected to sepsis work up if they had: maternal fever within 7 days before delivery or foul smelling liquor or prolonged rupture of membranes (>18 h) |
| Culture-negative sepsis (Possible bloodstream infection) | Baby has ALL of the following:   1. Any one of the clinical sign/symptoms as enlisted above   OR  Existence of predisposing *risk factors*: maternal fever within 7 days before delivery or foul smelling liquor or prolonged rupture of membranes (>18 h)  OR  Radiological evidence of *pneumonia*  OR  Positive septic screen (see “Notes”)   1. Blood culture not done or no organisms detected in blood 2. Physician institutes appropriate treatment for sepsis | Positive septic screen consists of two of the following parameters, namely total leukocyte count (TLC) <5 000 mm^3^, absolute neutrophil count <1 500 mm^3^, immature -to-total (IT) polymorph ratio of > 0·2, C-reactive protein >6 mg/dL, and micro-ESR > fall in mm more than 15 mm |
| Meningitis | This must meet ANY ONE of the following criteria   1. CSF culture is positive   AND  Baby has at least 1 of the signs or symptoms (as enlisted above)   1. If culture is negative, *all* of the following:   Any one of the clinical sign/symptoms listed above  AND  Any one of a–b:   1. Positive CSF examination with increased white cells, elevated protein, and/or decreased glucose 2. Positive gram stain for CSF   AND  If diagnosis is made antemortem, physician institutes appropriate antimicrobial therapy | \| CSF components \| Normal range \| \| --- \| --- \| \| Cells/mm^3^ \| Up to 30 cells \| \| PMN (%) \| 60% \| \| CSF protein (mg/dl) \| Up to 150 \| \| CSF/blood glucose (%) \| >60% \|   (PMN: polymorphonuclear leukocytes; CSF: cerebrospinal fluid) |
| Necrotizing enterocolitis | This must meet ALL of the following:   1. At least two of these signs/symptoms: Pre-feed gastric aspirate of >50% of previous feed, abdominal distension, vomiting, bile/blood-stained aspirate 2. Bloody stools or occult blood in the stools 3. At least one of the following radiological evidence: 4. Pneumatosis intestinalis 5. Portal air 6. Free air under the diaphragm (pneumoperitoneum) 7. Unchanging “rigid” loops of small bowel |  |
| Urinary tract infection | This must meet ANY ONE of the following criteria:   1. A positive urine culture ≥10^5^ microorganisms per cubic centimeter of urine with no more than two species of microorganisms   AND  At least 1 of the signs or symptoms (see “Notes”) with no other recognized cause   1. At least 1 of the signs or symptoms with no other recognized cause   AND  At least 1 of the following:   - Positive dipstick for leukocyte esterase and/or nitrate - Pyuria (urine specimen with ≥10 WBC/mm^3^ or ≥3 WBC/high-power ﬁr ≥ of unspun urine) - At least 2 urine cultures with repeated isolation of the same uropathogen (gram-negative bacteria or *S saprophyticus*) with ≥10^2^ colonies/ml in nonvoided specimens - ≤10^5^colonies/ml of a single uropathogen (gram-negative bacteria or *S saprophyticus*) in a patient being treated with an effective antimicrobial agent for a urinary tract infection - Physician institutes appropriate therapy for a urinary tract infection | *Symptoms:* Fever, lethargy, vomiting, irritability/failure to thrive,  *Signs:* Temperature (>37·5 °C or <36·5 °C), heart rate (>180 min or <100 min), apnea  Culture reports of urine sample only collected by suprapubic method is to be included in this study (culture reports of urine samples collected by bag or catheterization are to be excluded for this study)· |
| Systemic fungal infection | Baby has ANY ONE of the following criteria   1. Blood culture positive for yeasts   AND  Physician institutes appropriate therapy for a fungal infection   1. ≥2 of the risk factors (as mentioned adjoining in the notes)   AND  The presence of budding yeast/hyphae , either in cerebrospinal fluid or urine  AND  Physician institutes appropriate therapy for a systemic fungal infection | The main risk factors for systemic fungal infection (candidiasis) are as follows:   1. Gestational age <32 wk 2. Previous fungal colonization (especially of the gastrointestinal tract) 3. Presence of central venous catheters 4. Prior use of parenteral nutrition and lipid emulsions 5. Intubation time >7 days 6. Hospitalization >7 days 7. Shock or coagulopathy 8. Exposure to >2 antibiotics or any of third-generation cephalosporins 9. Exposure to systemic corticosteroids 10. Exposure to H2 blocker 11. Exposure to theophyllines |
| New episode of sepsis | When the neonate became symptomatic after 48 h of stopping appropriate antibiotic therapy or at clinician’s discretion if new organism was cultured at fresh deterioration in an ongoing episode. |  |

*Sepsis work up: Blood- Sepsis screen [1. Total leukocyte count (TLC), 2. Absolute neutrophil count,

3. Immature -to-total (IT) polymorph ratio, 4. Serum C-reactive protein level (semi-quantitative) and 5. Micro-ESR > fall in mm more than 15 mm] and culture (minimum volume of 0.5-1ml); CSF-Total and differential cell count, Biochemistry (sugar, and protein), Gram stain, and culture; urine sample- (whenever indicated) Gram stain, leukocyte count in unspun urine, and culture; other body fluid (whenever indicated)- Gram stain, leukocyte count, culture; radiology - chest, abdomen or other area (whenever indicated)

**Table B Definition of groups***

| Culture-positive sepsis groups | Place of delivery | Whether previously hospitalized or not | Age at admission | Admission to diagnosis interval |
| --- | --- | --- | --- | --- |
| ‘Community acquired infection’ (CAI) | Home | N | any | < 48 h |
| ‘Possibly community acquired infection’ (pCAI) | Health facility | N | > 168 h | < 48 h |
| ‘Possibly health–care associated infection’ (pHAI) | Health facility | N | < 168 h | < 48 h |
|  | any | Y | any |  |
| ‘Health–care associated infection’ (HAI) | any | any | any | > 48 h |

***** (all neonates in the groups were diagnosed before 48 h of admission except for HAI- diagnosed after 48h of admission)

**Table C: Quality assurance measures**

| Clinical | - Prospective data collection (research nurses) and checking the case record forms (CRF) daily for accuracy and completion (research physician) - Cross-checking of randomly selected 20% CRF at each site were checked by the team form the coordinating centre (faculty investigator and research physician) on a weekly basis - Prospective assignment of final diagnosis by two pediatricians (faculty investigator and research physician) |
| --- | --- |
| Microbiology | - Uniform, specially procured high-quality culture media [5% sheep blood agar (BioMerieux, France) and McConkey agar (Oxoid, Hampshire, the UK)] and antibiotics disks - Cross-checking of antibiotic disk batches using ATCC strains on a regular basis (research microbiologist) - Validating identification and antibiograms of 10% isolates from each site at a different site (external quality assurance scheme; EQAS) |
| Data management | - Online double data entry in real-time at the sites (Data entry operators) - Discrepancies in double data entry resolved on an ongoing basis (research programmer) - Three-monthly data audits; errors resolved in consultation with site investigators (research biostatistician) |
| Overall | - Training of all research nurses off- and on-site using SOPs and videos (e.g., sample collection) initially and at periodic intervals (faculty investigator and research physician) - Dry run for four weeks before finalization of the CRF and SOPs - Weekly review meetings for assessing the progress |

**Table D: Additional demographic details**

| Items | Overall  (n =2588) |
| --- | --- |
| Birth weight^#^ (n= 2058) | 2204 ±731 |
| <1000 | 89 (4.3%) |
| 1000-1499 | 295 (14.3%) |
| 1500-2499 | 816 (39.6%) |
| ≥2500 | 858 (41.7%) |
| Gestational age^#^ (n=2583) | 35.4±2.8 |
| ≤27 | 25 (1.0%) |
| 28-31 | 295 (11.4%) |
| 32-36 | 767 (29.7%) |
| ≥ 37 | 1496 (57.9%) |
| Age at admission (days) |  |
| <1 | 573 (22.2%) |
| 1-7 | 1097 (42.5%) |
| ≥8 | 914 (35.4%) |

**Table E: Birth weight and gestational age specific prevalence rates of infections**

|  | | Total sepsis | Culture positive | Meningitis | Systemic fungal  Infections | Necrotizing enterocolitis |
| --- | --- | --- | --- | --- | --- | --- |
| Birth weight specific (g) | | | | | | |
|  | N=2058 | (n=1117) | (n=263) | (n=132) | (n=79) | (n=104) |
| >=2500 | 858 | 384 (34.4%) | 83 (31.6%) | 38 (28.8%) | 16 (20.3%) | 16 (15.4%) |
| 1500-2499 | 816 | 453 (40.6%) | 111 (42.2%) | 46 (34.9%) | 33 (41.8%) | 30 (28.9%) |
| 1000-1499 | 295 | 204 (18.3%) | 50 (19.0%) | 34 (25.8%) | 17 (21.5%) | 36 (34.6%) |
| <1000g | 89 | 76 (6.8%) | 19 (7.2%) | 14 (10.6%) | 13 (16.5%) | 22 (21.1%) |
| Gestation (weeks) | | | | | | |
|  | N=2583 | (n=1413) | (n=338) | (n=180) | (n=91) | (n=113) |
| 37 and above | 1496 | 742 (52.5%) | 173(51.2%) | 82(45.6%) | 36 (39.6%) | 25 (22.1%) |
| 32-36 wk | 767 | 430 (30.4%) | 105(31.1%) | 57 (31.7%) | 31 (34.1%) | 34 (30.1%) |
| 28-31 wk | 295 | 220 (15.6%) | 55(16.3%) | 41 (22.8%) | 20 (22.0%) | 49 (43.4%) |
| <= 27 wk | 25 | 21 (1.5 %) | 5 (1.5%) | 0 | 4 (4.4%) | 5 (4.4%) |

**Table F: Admission to diagnosis interval**

|  | Total sepsis  (n=1409*) | Culture positive sepsis  (n=339) |
| --- | --- | --- |
| ≤24 h | 1320 (93.6%) | 317 (93.5%) |
| Day 2 | 22 (1.6%) | 6 (1.2%) |
| Day 3 | 9 (0.6%) | 2 (0.6%) |
| Day 4 | 4 (0.3%) | 2 (0.6%) |
| Day 5 | 1 (0.1%) | 0 |
| Day 6 | 4 (0.3%) | 0 |
| Day 7 | 2 (0.1%) | 0 |
| Day 8 or more | 47 (3.3%) | 12 (3.5%) |

*Age at onset missing for 7 neonates having culture-negative sepsis for whom cultures could not be taken.

**Table G: Outcomes compared by place of delivery**

| Items | Delivered at home (n=550) | Delivered at hospital /other places^$^ (n=2038) |
| --- | --- | --- |
| Total sepsis | 325 (59.1%) | 1091 (53.5%) |
| Culture positive sepsis | 75 (13.6%) | 264 (12.9%) |
| Culture negative sepsis | 250 (45.4%) | 827 (40.6%) |
| Meningitis | 45 (8.2%) | 135 (6.6%) |
| Necrotizing enterocolitis | 16 (2.9%) | 97 (4.8%) |
| Systemic fungal infection | 11 (2.0%) | 79 (3.9%) |
| Others* | 3 (0.5%) | 10 (0.5%) |
| All-cause mortality  Primary cause of death  Infections  Prematurity  Perinatal asphyxia  Malformations  Others | 51 (9.3%)  38 (74.5%)  7 (13.7%)  3 (5.9%)  1 (1.9%)  2 (3.9%) | 192 (9.4%)  115 (59.9%)  30 (15.6%)  6 (3.1%)  17 (8.8%)  24 (12.5%) |
| Case fatality rates, n (%)  Total sepsis  Culture positive  Culture negative  Meningitis  Necrotizing enterocolitis  Systemic fungal infection | 37/325 (11.4%)  20/75 (26.7%)  17/250 (6.8%)  4/45 (8.9%)  5/16 (32.2%)  3/11 (27.3%) | 114/1091 (10.4%)  58/264 (22.0%)  56/827 (6.8%)  18/135 (13.3%)  15/97 (15.5%)  17/79 (21.5%) |

Data expressed as no (%);$ others include four neonates who were brought to index hospital as ‘Unknown’ and 16 who were born on the way to hospital or at its periphery. *Bone/joint infection (n=9), urinary tract infection (n=4)

**Table H: AMR in Gram positive pathogens**

|  | *S aureus* | | | | *S epidermidis* | | | | *S hemolyticus* | | | | *E faecium* | | | |
| --- | --- | --- | --- | --- | --- | --- | --- | --- | --- | --- | --- | --- | --- | --- | --- | --- |
|  | 1 (n=5) | 2 (n=6) | 3 (n=6) | 4 (n=2) | 1 (n=4) | 2 (n=2) | 3 (n=9) | 4 (n=2) | 1 (n=1) | 2 (n=3) | 3 (n=8) | 4 (n=0) | 1 (n=1) | 2 (n=5) | 3 (n=6) | 4 (n=0) |
| Methicillin | 1/ 5 (20) | 3 /6 (50) | 1/ 6 (16.7) | 1/ 2 (50) | 3 /4 (75) | 2 /2 | 8 /9 (88.9) | 2/ 2 | 1 /1 | 3/ 3 | 8/8 | - | - | 1 /1 | 1 /2 (50) | - |
| Amoxycillin | 5/ 5 | 5 /6 (83.3) | 56/6 | 1 /2 (50 ) | 3 /3 | 2/ 2 | 6 /9 (66.7 ) | 2 /2 | 1 /1 | 3 /3 | 8/8 | - | - | 2 /4 (50) | 5 6/6 | - |
| Amoxycillin + clavulanate | 1 /5 (20) | 3/ 6 (50) | 1/ 5 (20) | 1 /2 (50) | 0 /3 | 2/ 2 | 5 /9 (55.6) | 2 /2 | 1 /1 | 2 /3 (66.7) | 7/7 | - | - | - | 1 /2 (50) | - |
| Amikacin | 1/ 5 (20) | 3 /6 (50) | 1 /6 (16.7) | 1/ 2 (50) | 0/ 3 | 1/ 2 (50) | 3/ 9 (33.3) | 1/ 2 (50) | 0/ 1 | 0 /3 | 8/8 | - | - | 1/ 1 | 1 /2 (50) | - |
| Vancomycin | 0 /5 | 0/ 6 | 1 /6 (20) | 0/ 2 | 0/ 4 | 0/ 2 | 0 /9 | 0/ 2 | 0/ 1 | 0 /3 | 0 /8 | - | 0 /1 | 2 /5 (40) | 3/6 (50) | - |
| Teicoplanin | 2/ 5 (40) | 1 /6 (16.7) | 0 /6 | 1 /2 (50) | 1 /3 (33.3) | 1/ 2 (50) | 2/ 9 (22.2) | 1 /2 (50) | 0/ 1 | 0/ 3 | 3/ 8 (37.5) |  | 0 /1 | 1 /5 (20) | 3 4/5 (80) | - |
| Gentamicin | 1 /5 (20) | 3/6 (50) | 1 /6 (16.7) | 1 /2 (50) | 0 /3 | 1 /2 (50) | 6 /9 (66.7) | 1/ 2 (50) | 1 /1 | 2 /3 (66.7) | 7/8 (87.5) |  | 1/ 1 | 3 /5 (60) | 45/6 (83.3) | - |
| Ciprofloxacin | 5/5 | 6/ 6 | 5 6/6 | 2 /2 | 2 /3 (66.7) | 1 /2 (50) | 7/9 (77.8) | 2 /2 | 1 /1 | 3/ 3 | 7/8 (83.3) |  | 1 /1 | 4/5 (80) | 56/6 | - |
| Linezolid | 1 /5 (20) | 0 /6 | 0 /6 | 0 /2 | 0 /3 | 0 /2 | 0/ 9 | 0 /2 | 0/ 1 | 0/3 | 0/ 8 |  | 0/ 1 | 0/ 5 | 0/ 6 | - |
| Netilmicin | 1 /5 (20) | 0 /4 | 0 /4 | 0/ 2 | 0/ 3 | 0/ 2 | 0/ 9 | 0/ 2 | 0/ 1 | 0/ 3 | 1/ 8 (12.5) |  | - | - | 0/ 2 | - |

Data expressed as n (%). *1= CAI, Community acquired Infection; 2= pCAI= Possibly Community acquired Infection;3= pHA, Possibly healthcare associated infection; 4= HAI,Healthcare associated infection*

**Table I: AMR in Gram negative pathogens**

|  | *A baumannii* | | | | *E  cloacae* | | | | *K pneumoniae* | | | | *E coli* | | | |
| --- | --- | --- | --- | --- | --- | --- | --- | --- | --- | --- | --- | --- | --- | --- | --- | --- |
|  | 1 (n=8) | 2 (n=2) | 3 (n=35) | 4 (n=0) | 1 (n=3) | 2 (n=4) | 3 (n=15) | 4 (n=0) | 1 (n=6) | 2 (n=4) | 3 (n=39) | 4 (n=1) | 1 (n=9) | 2 (n=4) | 3 (n=17) | 4 (n=2) |
| Cefotaxime | 8/ 8 (100 ) | 2/ 2 | 33/35 (94.3 ) | **-** | 2/3 (66.7) | 2/ 3 (66.7) | 13 /15 (86.7) | **-** | 4/ 6 (66.7) | 4/ 4 (100) | 33/37 (89.2 ) | 1/1 | 6 /9 (66.7 ) | 3/ 4 (75 ) | 13 /16 (81.2 ) | 2/ 2 |
| Gentamicin | 7/8 (87.5) | 2 /2 | 30/35 (85.7) | **-** | 2/3 (66.7) | 2/ 4 (50) | 12 /16 (75) | **-** | 4 /6 (66.7 ) | 4/4 (100) | 31/39 (79.5 ) | 1 /1 | 3 /9 (33.3 ) | 1/ 4 (25 ) | 10/ 17 (58.8 ) | 1 /2 |
| Amikacin | 6/8 (75) | 1 /2 (50) | 29/36 (80.5 ) | **-** | 2/3 (66.7) | 2/4 (50) | 9 /16 (56.2) | **-** | 4/ 6 (66.7) | 4 /4 (100) | 27/39 (69.2 ) | 1/ 1 | 1 /9 (11.1 ) | 1 /4 (25 ) | 7/ 17 (41.2) | 0/ 2 |
| Ciprofloxacin | 7/8 (87.5) | 2 /2 | 32/36 (88.9 ) | **-** | 2/3 (66.7) | 2 /4 (50) | 8 /16 (50 ) | **-** | 3 /6 (50 ) | 1/ 4 (25 ) | 27/39 (69.2 ) | 1 /1 | 8 /9 (88.9 ) | 2 /4 (50 ) | 12/ 17 (70.6 ) | 1/ 2 |
| Piperacillin-tazobactum | 7/8 (87.5) | 2 /2 | 34/35 (97.1 ) | **-** | 2/3 (66.7) | 3/4 (75) | 12 /16 (75 ) | **-** | 4/ 6 (66.7) | 4 /4 (100) | 30/39 (76.9 ) | 1 /1 | 3 /9 (33.3 ) | 1 /4 (25 ) | 12/17 (70.6) | 1/ 2 |
| Meropenem | 7/8  (87.5) | 2/ 2 | 32/36  (88.9 ) | **-** | 2/3  (66.7) | 2 /4  (50) | 9/ 16  (56.2 ) | **-** | 4/ 6  (66.7) | 4/ 4  (100) | 26/39  (66.7) | 1/ 1 | 0 /9  (0 ) | 1/ 4  (25 ) | 9/17  (52.9) | 1 /2 |
| Cefoperazone-sulbactum* | 6/8  (75) | 2 /2 | 27/36  (75 ) | **-** | 2/3  (66.7) | 2 /4  (50) | 9 /16  (56.2 ) | **-** | 4/ 6  (66.7) | 4 /4  (100) | 28/39  (71.8 ) | 1/ 1 | 2 /9  (22.2 ) | 1 /4  (25 ) | 10/17  (58.8 ) | 1 /2 |
| Colistin | 0 /8 | 0/ 2 | 1 /35  (2.9 ) |  | 0/ 3 | 0 /4 | 0 /16 | **-** | 0 /6 | 0 /4 | 0 /39 | 0/ 1 | 0 /9 | 0 /4 | 0 /16 | 0 /2 |

Data expressed as n (%).

*1= CAI, Community acquired Infection; 2= pCAI= Possibly Community acquired Infection;3= pHA, Possibly healthcare associated infection; 4= HAI,Healthcare associated infection; adapted EUCAST guidelines*

**Table J: Antimicrobial resistance pattern of fungal organisms**

|  |  | Total |
| --- | --- | --- |
| *Candida albicans*  (n=20) | Fluconazole | 0/20 |
|  | Amphotericin B | 0/20 |
|  | Voriconazole | 0/17 |
| *Candida tropicalis*  (n=17/20) | Fluconazole | 0/17 |
|  | Amphotericin B | 0/17 |
|  | Voriconazole | 0/17 |
| *Candida parapsilosis*  (n=16/18) | Fluconazole | 0/16 |
|  | Amphotericin B | 0/16 |
|  | Voriconazole | 0/16 |
| *Candida krusei*  (n=14/16) | Fluconazole | 13/14 (92.9%) |
|  | Amphotericin B | 2/14 (14.3%) |
|  | Voriconazole | 1/12 (8.3%) |
| *Candida glabrata*  *(n=8)* | Fluconazole | 7/8 (87.5) |
|  | Amphotericin B | 0/8 |
|  | Voriconazole | 0/7 |
| *Candida pelliculosa*  (n=8/9) | Fluconazole | 0/8 |
|  | Amphotericin B | 0/8 |
|  | Voriconazole | 0/8 |
| Other Candida spp  (n=9/10) | Fluconazole | 1/9 (11.1) |
|  | Amphotericin B | 2^#^/9 (22.2) |
|  | Voriconazole | 1/8 (12.5%) |

*^#^2 strains of C guilliermondii*

**Table K: Pathogen specific case fatality rates**

| Pathogen | Overall | CAI | PCAI | PHAI | HAI |
| --- | --- | --- | --- | --- | --- |
| *Acinetobacter baumannii* | 17/46 (37.0%) | 2/8 (25.0%) | 0/2 | 15/36 (41.7%) | - |
| *Klebsiella pneumoniae* | 10/50 (20.0%) | 2/6 (33.3%) | 0/4 | 7/39 (17.9%) | 1/1 |
| *E coli* | 13/32 (40.6%) | 5/9 (55.0%) | 0/4 | 7/17 (41.2%) | 1/2 |
| *Enterobacter cloacae* | 3/23 (13.0%) | 1/3 | 1/4 | 1/16 | - |
| *Enterococcus faecium* | 3/12 (25.0%) | 0/1 | 2/5 (40.0%) | 1/6 | - |
| *Staphylococcus aureus* | 3/19 (15.8%) | 0/5 | 1/6 | 2/6 | 0/2 |
| *Staphylococcus epidermidis* | 2/17 (11.8%) | 0/4 | 0/2 | 1/9 | 1/2 |
| *Staphylococcus hemolyticus* | 3/13 (23.0%) | 1/1 | 2/3 | 0/9 | - |
| *Staphylococcus hominis* | 1/7 (14.3%) | 1/2 | 0/2 | 0/3 | - |
| *Candida albicans* | 5/20 (25.0%) | - | - | 5/18 | 0/2 |
| *Candida tropicalis* | 3/20 (15.0%) | 1/1 | 0/1 | 1/15 | 1/3 |
| *Candida krusei* | 3/16 (18.7%) | 0/1 | 0/2 | 3/13 (25.0%) | - |
| *Candida parapsilosis* | 6/18 (33.3%) | 0/1 | 1/3 (33.3) | 4/13 (30.8%) | 1/1 |
| *Candida pelliculosa* | 2/9 (2.20%) | - | 0/1 | 2/8 | - |
| *Candida glabrata* | 2/8 (25.0%) | - | 1/1 | 1/7 | - |
| Other *Candida* spp | 4/10 | - | 1/1 | 3/7 | 0/2 |

*CAI, Community acquired Infection; pCAI= Possibly Community acquired Infection; pHAI, Possibly healthcare associated infection; HAI,Healthcare associated infection*

**S1 file panel A: Clinical SOP algorithm**

**Steps**

**By whom?**

**Research nurse/resident** (whoever takes the blood sample); Supervised by **RO**

**Research nurse/resident** (whoever takes the blood sample); Supervised by **RO**

Requisition form: **Research nurse**

Form C: **Research officer**

**Laboratory attendant;** supervised by **research nurse**

**If sepsis is suspected**

**Data entry operator;** supervised by **RO**

Form A: **Research nurse/RO (clinical)**

Form C: **RO (microbiology)**

Form A: **Research nurse**

Form C: **RO (clinical and microbiology)**

**Research nurse;** supervised by **RO**

Data entry

Transport of samples along with the forms to the site laboratory

Collecting and filing the reports

Updating Form A and Form C based on the laboratory reports and case-files

Handing over the filled and updated forms to DEO

Filling-up the requisition forms and microbiology form- Form C

Incubating the sample at 37^0^C

Labeling the samples (particularly blood culture bottle)

**Resident/research nurse;** supervised by site PI/co-PI

Sepsis screen: To be done and interpreted by the **resident/research nurse**

**Investigations to rule out/rule-in sepsis**

- Blood/ CSF culture
- Sepsis screen, Gram stain, CSF biochemical analysis
- Others (e.g. chest X-ray) as indicated

**Research nurse;** supervised by **RO**

20% entries to be checked by RO

Record daily the nature of interventions and feeding

*(Fill-up pages 2-3 of Form A)*

**Research nurse;** supervised by **Research Officer** (**RO)**

**Track all babies admitted in the NICU in the last 24 hours**

- Individually track all babies using a register
- Collect baseline information and fill-up case record form - Form A ( page 1)

**Explanation to S1 file panel A:**

*Identification and enrolment of subjects*

- Enrolment will be carried out on all weekdays including Sundays
- The research nurse would start tracking the new babies admitted in the NICU during their shift time. In addition, nurses posted in the morning shift would also collect information for infants admitted in the previous night (before 0800 hours).
- The nurses would maintain a register to record the details of all the babies admitted in the NICU
- Research officer (RO) shall supervise enrolment on a daily basis The RO would counter-check the register and forms daily and affix his/her signature
- The nurse(s) would also attend the NICU clinical rounds to collect information about babies suspected to have sepsis
- Immediate management of the infants (feeding, method of feeding – oral vs. parenteral, monitoring for complications other than sepsis, etc.) would be based on the units’ existing protocol.
- Each baby would be followed-up until discharge from the hospital

*Baseline data collection in NICU*

- Research nurse will retrieve the baseline information from the case files (and fill-up the page 1 of case record form- Form A); in case of any query, she will contact the concerned resident doctor.
- Research officer (RO) shall supervise data retrieval on a daily basis

*Monitoring the babies for sepsis*

- The research nurse would attend the clinical rounds of the Consultant
- She would go through the case files of each baby after the rounds and fill up the relevant information
- The research nurse would update the daily monitoring/screening form (pages 2 and 3 of Form A) for sepsis every 24 hours until the baby is shifted to the ward or discharged from the hospital; this form would be counter-signed by the concerned RO daily
- A uniform protocol will be followed for screening the babies for sepsis on the basis of maternal risk factors (in case of EOS) or clinical symptoms/signs (EOS or LOS).

*For those infants suspected to have sepsis*

- The nurse/resident doctor would draw the blood samples for culture, screen, and other investigations (see section below); she will inform the RO before collecting the samples
- The RO (Clinical) would fill microbiology requisition form- Form C and then handover it to the microbiology team
- The research nurse would help the resident/duty staff in emergency management like initiation of oxygen therapy, treatment of seizures, etc.
- Further clinical management including antibiotic therapy, ordering investigations like X-ray chest/abdomen, CSF examination etc. would be decided by the clinical team

*Collection, labeling, and storage of blood/CSF samples*

- Blood, CSF, and other cultures would be collected as per standard guidelines (see https://www.youtube.com/watch?v=1VsBBn2quTc and/or http://www.newbornwhocc.org/Skill-videos.html)
- The nurse would label the samples and keep in the incubator immediately after collection; the blood sample collected by the resident (e.g. in the night hours) would also be kept in the incubator. They should NOT be refrigerated.
- The CSF sample should be immediately sent to the microbiology laboratory. The research nurse should co-ordinate with the laboratory attendant to ensure that the sample is sent to the laboratory immediately.
- A uniform label would be used for all the samples at all sites. The labels would be sent from the nodal (NHKC, AIIMS) office to the microbiology lab. The RO would ensure that these labels are pasted in the culture bottles sent to the nursery/NICU.
- The nurse would fill up the requisition forms and hand over the samples to the lab attendant twice daily (at 10 AM and 3 PM)

*Transport of samples and collecting the reports*

- The laboratory attendant would transport the samples from the wards/ICU to the microbiology lab twice daily (except on Sundays) – at 10 AM and 4 PM; on Saturdays, the samples would be sent once at 11 AM
- The lab attendant would also handover the reports from the laboratory to the research nurse(s)

*Updating the proforma and handing over to data entry operator (DEO)*

- The research nurse would update the Form A of each baby daily until they are discharged from the hospital (or until their death)
- The research nurse would hand over the completed Form A to the DEO after getting countersigned by the RO within 24 hours of discharge/death
- The research nurse would maintain a register to keep record of the forms that are being sent to DEO; she should get the signature of the DEO in the register for the forms that are dispatched to him/her
- The RO (clinical) would make sure that the Form C is filled up by RO (micro) and handed over to DEO
- Quality check: RO would fill at least one form independently - for a baby randomly picked by him/her or the PI – every day. He/she would then cross-check the form filled by the nurse for the same baby and give the feedback to the nurse
- All the completed forms would be counter-signed by the RO (clinical) and then by site PI/co-PI (clinical)

*Data entry*

- The data entry operator (DEO) posted at each site would enter the data in the database on a daily basis
- Double data entry: All the proformas would be doubly entered by exchanging among DEO other than who did the first entry (at the same or another site in the DeNIS collaboration).
- The DEO should maintain a register to keep record of the forms that are being sent to other DEO for double data entry. The latter should sign this register upon receiving the forms

*Storage of samples and transport to reference laboratory*

- Each microbiology laboratory would store the positive cultures in their respective laboratories for a period of 7 days; the isolates should be sent to the reference laboratory (AIIMS) on each Friday at 2 PM
- The laboratory attendant would transport the samples from the respective sites to AIIMS. He/she would handover the samples to Dr Deepali / Ms Neelam in HIC lab, AIIMS. In case a particular Friday is a Government holiday, the samples would be sent on Thursday 2 PM.

The contingency grant provided to the microbiology PIs will be utilized to pay for the local travel of the lab attendant (by public transport).

**S1file panel B: External Quality Assurance Scheme (EQAS)**

**Characterization of errors** (CDC, Atlanta, USA recommendations)**:**

The comparison of disc diffusion results between the reference and the site labs are interpreted as per the following (Adopted form Manual of Clinical Microbiology, 7^th^ edition):

**Minor error** (S - sensitive, I – intermediate, R- resistant)**:**

> 3 mm but interpretation still correct

> 3 mm interpretation changes from S I

>3 mm interpretation changes from I S

>3 mm interpretation changes from R I

>3 mm interpretation changes from I R

**Major error:**

>3 mm and interpretation changes from S R (over-calling resistance)

**Very major error:**

>3 mm and interpretation changes from R S (under-calling resistance)

**Acceptability:**

Minor error is acceptable

Major error and intermediate error acceptance limit is <5%

Very major error: none

The laboratory that undertook the quality control steps is participating in the national EQAS of Medical Microbiology.

For this study site, we performed EQAS in 2 phases.

In first phase, 100% samples were screened for outborn site for consecutive three months to rule out any ambiguity.

Of the 82 isolates checked from the stocked samples, 2 could not be revived or had mixed growth; so, 80 were available for cross-checking. Of these, 75/80 (93.75%) were confirmed. None was found to be different at genus level; however, there few (6.25%) were found to be discrepant in identity at species level only. Out of 80, 73/80 (91.2%) were concordant in AST. Only 8.7% were discrepant in AST (having major or minor variations).

In second phase (rest of the study period), 10% isolates were randomly selected from the 4 groups as follows: 10% gram negative organism (comprising of 3 most commonest gram negative bugs), 10% gram positive organism (comprising of 2 most commonest gram positive bugs), 10% rare organism (isolated less commonly), and 10% organism having rare AST (unusual pattern). A total of 25 samples were cross checked from the stocked samples. Of these, one each could not be revived and had mixed growth. Of the remaining 23 isolates, 100% were confirmed in identity. With regards to AST, 91.3% were concordant in AST. There was no major discrepancy and only two had minor discrepancy, which were acceptable.

All the error were checked and rectified before inclusion into the study for this manuscript

.

**S1 file references**

1. Horan TC, Andrus M, Dudeck MA· CDC/NHSN surveillance definition of health care-associated infection and criteria for specific types of infections in the acute care setting· *Am J Infect Control* 2008;**36:**309–32.
