# Supplementary material for: Alarming rates of antimicrobial resistance and fungal sepsis in outborn neonates in North India
Source: PLoS One. 2018 Jun 28;13(6):e0180705. doi: 10.1371/journal.pone.0180705 (PMC6023165; doi:10.1371/journal.pone.0180705)
Supplement: S3 File — (DOCX) [file pone.0180705.s003.docx]

**Investigators of the Delhi Neonatal Infection Study (DeNIS) collaboration**

**Chacha Nehru Bal Chikitsalaya, New Delhi**

*Clinical:* Mamta Jajoo (PI), Vikas Dabbas (co-PI), Priya Sebastian, Jincymol George, Steffi Wilson, Dhanya Alex, Anchal Wadhwa

*Microbiology:* Vikas Manchanda (PI), Hitender Gautam (co-PI), Deepika Gupta, Manisha Jain, Abhinav Kher, Zulfikar Ali Bhuttoo, Ashish Kumar, Ashwani Kumar

**Vardhman Mahaveer Medical College and Safdarjung Hospital, New Delhi**

*Clinical:* KC Aggarwal (PI, 2011–14), MS Prasad (PI, 2010–11), Harish Chellani (PI, 2010-14), Sugandha Arya (co-PI), Sumita Saluja, Vidya Kumari, Asha Susan Philip, Simi P Johny, Rosemol T. Matchachan, Vandana Gulati, Mary Abraham, Sony Varghese, Mitali Bhardwaj, Jenifer Chinnu Abraham, Ann Rainu Jose, Nisha Rani, Longjam Shilhenba Meitei

*Microbiology:* Monorama Deb (PI), Rajni Gaind (co-PI), Manjari Choudhary, Charu, Kunj Bihari Gupta, Mamta Dhoopar, Deepak Kumar, Vandana Rani, Shubh Darshan

**Maulana Azad Medical College and LNJP Hospital, New Delhi**

*Clinical:* Siddrath Ramji (PI), Manoj Modi, Neeraj Gupta, Ashish Jain, Anil Mor, Nidhi Goel, Benu Varghese, Dona Jose, Honey Mary James, Mary Abraham, Vimala D, Rimjim J, Mariya Thomas, Simi Thomas, Manish Sethi

*Microbiology****:*** KS Prakash (PI), Surinder Kumar (site co-PI), Jogender Singh Nim, Ashok Kumar, Madan Singh

**All India Institute of Medical Sciences (AIIMS), New Delhi (Nodal Center)**

*Clinical:* Ashok Kumar Deorari (PI), Ramesh Agarwal (co-PI), M Jeeva Sankar (co-PI), Manju Saksena (co-PI), Anu Thukral (co-PI), Suman Chaurasia, Shuchita Gupta, Deeksha Mittal, Kaustav Banerjee, Anjali Thareja, Sreemol Sarasan, Angel George, Shinsa John, Seena KS, Suman Singh, Joshmi Philip, Annai Therasa D., Pratibha Gupta, Akhilesh Kumar Sharma, Shobhit Sharma, Ravi Kumar, Sant Lal, Meenakshi

*Microbiology:* Arti Kapil (PI), Poorva Mathur P (co-PI), Deepali Agarwal, Neelam Manral, Shyam Narayan Yadav

*Biostatistics:* Sreenivas Vishnubhatla, Chander Prakash Yadav CP, SS Suresh, Kaustav Banerjee

**Indian Council for Medical Research (ICMR):** Reeta Rasaily, Malabika Roy, Vasantha Muthuswamy, Anju Sinha

**Project PI and team leader:** Vinod K Paul

**Author contributions**

**Conceptualization (in alphabetical order):** Ramesh Agarwal Kailash C Aggarwal Sugandha Arya Suman Chaurasia Harish Chellani Monorama Deb Ashok K Deorari Rajni Gaind Mamta Jajoo Arti Kapil Surinder Kumar Vikas Manchanda Vinod K Paul Siddharth Ramji Reeta Rasaily Krishnaprakash S M Jeeva Sankar Vishnubhatla Sreenivas Poorva Mathur Hitender Gautam SS Suresh Chander Prakash Yadav Deepika Gupta Deeksha Mittal

**Data curation:** SS Suresh Chander Prakash Yadav Deeksha Mittal Suman Chaurasia M Jeeva Sankar Vishnubhatla Sreenivas Mamta Jajoo Vikas Manchanda Deepika Gupta Ramesh Agarwal

**Formal analysis:** Chander Prakash Yadav Suman Chaurasia M Jeeva Sankar Mamta Jajoo Vikas Manchanda Hitender Gautam SS Suresh Ramesh Agarwal Vishnubhatla Sreenivas Sugandha Arya Harish Chellani Siddhartha Ramji Kailash C Aggarwal Arti Kapil Rajni Gaind Monorama Deb Surinder Kumar Krishnaprakash S Poorva Mathur Reeta Rasaily Ashok K Deorari Vinod K Paul

**Funding acquisition:** Vinod K Paul Ashok K Deorari Ramesh Agarwal M Jeeva Sankar

**Investigation:** *Clinical:* Mamta Jajoo Deeksha Mittal Suman Chaurasia M Jeeva sankar

*Microbiology:* Vikas Manchanda Hitender Gautam Deepika Gupta

**Methodology:** Mamta Jajoo, Vikas Manchanda Suman Chaurasia M Jeeva Sankar Ramesh Agarwal Sugandha Arya Harish Chellani Kailash C Aggarwal Siddharth Ramji Monorama Deb S Krishnaprakash Rajni Gaind Surinder Kumar Sreenivas Vishnubhatla Arti Kapil Poorva Mathur Ashok K Deorari Vinod K Paul

**Project administration:** Vinod K Paul Ramesh Agarwal M Jeeva Sankar

**Resources:**

**Software:**

**Supervision:** Mamta Jajoo Vikas Manchanda Vinod K Paul Ramesh Agarwal M Jeeva Sankar

**Validation:**

**Visualization:**

**Writing (original draft preparation):** Suman Chaurasia M Jeeva Sankar Ramesh Agarwal Mamta Jajoo Vikas Manchanda Vishnubhatla Sreenivas Chander Prakash Yadav Vinod K Paul Deeksha Mittal SS Suresh Deepika Gupta

**Writing (review and editing):** M Jeeva Sankar Suman Chaurasia Mamta Jajoo Vikas Manchanda Ramesh Agarwal Harish Chellani Siddharth Ramji Sugandha Arya Vishnubhatla Sreenivas Arti Kapil Reeta Rasaily Ashok K Deorari Chander Prakash Yadav Hitender Gautam Rajni Gaind Poorva Mathur Surinder Kumar Kailash Aggarwal Vinod K Paul and Investigators of the *De*lhi *N*eonatal *I*nfection *S*tudy (DeNIS) collaboration, New Delhi, India
